# Evolutionary conservation of sequence and secondary structures in CRISPR repeats

**DOI:** 10.1186/gb-2007-8-4-r61

**Published:** 2007-04-18

**Authors:** Victor Kunin, Rotem Sorek, Philip Hugenholtz

**Affiliations:** 1 DOE Joint Genome Institute, 2800 Mitchell Drive, Walnut Creek, CA 94598, USA

## Abstract

**Background:**

Clustered regularly interspaced short palindromic repeats (CRISPRs) are a novel class of direct repeats, separated by unique spacer sequences of similar length, that are present in approximately 40% of bacterial and most archaeal genomes analyzed to date. More than 40 gene families, called CRISPR-associated sequences (CASs), appear in conjunction with these repeats and are thought to be involved in the propagation and functioning of CRISPRs. It has been recently shown that CRISPR provides acquired resistance against viruses in prokaryotes.

**Results:**

Here we analyze CRISPR repeats identified in 195 microbial genomes and show that they can be organized into multiple clusters based on sequence similarity. Some of the clusters present stable, highly conserved RNA secondary structures, while others lack detectable structures. Stable secondary structures exhibit multiple compensatory base changes in the stem region, indicating evolutionary and functional conservation.

**Conclusion:**

We show that the repeat-based classification corresponds to, and expands upon, a previously reported CAS gene-based classification, including specific relationships between CRISPR and CAS subtypes.

## Abstract 2

The categorisation and structural analysis of Clustered Regularly Interspaced Short Palindromic Repeats (CRISPRs) sequences from 195 microbial genomes show that repeats from diverse organisms can be grouped based on sequence similarity, and that some groups have pronounced secondary structures with compensatory base changes.

## Background

Clustered regularly interspaced short palindromic repeats (CRISPRs) are repetitive structures in Bacteria and Archaea composed of exact repeat sequences 24 to 48 bases long (herein called repeats) separated by unique spacers of similar length (herein called spacers) [1,2]. The CRISPR sequences appear to be among the most rapidly evolving elements in the genome, to the point that closely related species and strains, sometimes more than 99% identical at the DNA level, differ in their CRISPR composition [3,4].

Up to 45 gene families, called CRISPR-associated sequences (CASs), appear in conjunction with these repeats and are hypothesized to be responsible for CRISPR propagation and functioning [2,5,6]. It has been proposed that CASs can be divided into seven or eight subtypes, according to their operon organization and gene phylogeny [5,6]. Phylogenetic analysis additionally indicates that CASs have undergone extensive horizontal gene transfer, as very similar CAS genes are found in distantly related organisms [6,7]. CRISPRs and CASs have been found on mobile genetic elements, such as plasmids, *skin *mobile elements, and even prophages, suggesting a possible distribution mechanism for the system [7-9].

CRISPRs have been suggested to play roles in replicon partitioning [1], DNA repair [10], regulation [5] and chromosomal rearrangement [11]. It was recently reported that the spacers are often highly similar to fragments of extrachromosomal DNA, such as phage or plasmid DNA [3,12]. It was suggested that the CRISPR/CAS system participates in an antiviral response, probably by an RNA interference-like mechanism. The proposed mechanism for this CRISPR function involves sampling and maintaining a record of invasive DNA elements, and inhibition of gene functions necessary for invasion [12]. Indeed, it was recently shown that CRISPRs provide acquired resistance against viruses in prokaryotes [13].

Despite in-depth analyses of CASs, the nature of the repeat sequences has not been examined closely. This is presumably because repeats, as short DNA sequences, have less comparative potential than protein-coding genes. Previous studies have noted only that repeats are highly variable, and do not appear to be similar between organisms [2,7]. However, we show that repeats from diverse organisms can be grouped into clusters based on sequence similarity, and that some clusters have pronounced secondary structures with compensatory base changes. We further show that there is a clear correspondence between CAS subtypes and repeat clusters. Our findings have important implications for CRISPR function and diversity.

## Results

To obtain a set of CRISPR arrays we employed the PILER-CR program [14] on 439 currently available bacterial and archaeal genomes in IMG version 1.50 [15]. We found 561 arrays, ranging in size from 3 to 220 repeats, in 195 genomes (44% of the genomes tested). These results are in agreement with the results of Godde *et al*. [7], who found CRISPR arrays in 40% of the genomes they tested. Overall, our set of CRISPRs contained 561 repeat sequences (as repeats are generally identical within an array) and 13,372 spacers.

Repeats were first noticed to be palindromic by Mojica *et al*. [16], a feature that was subsequently incorporated into the acronym CRISPR [2]. We hypothesized that the palindromic signature might be indicative of a functional RNA secondary structure within the repeat. This hypothesis is supported by the experimental demonstration that CRISPRs are transcribed and processed into non-messenger RNAs in several Archaea [17], indicating that they are active through an RNA intermediate.

**Figure 1:**
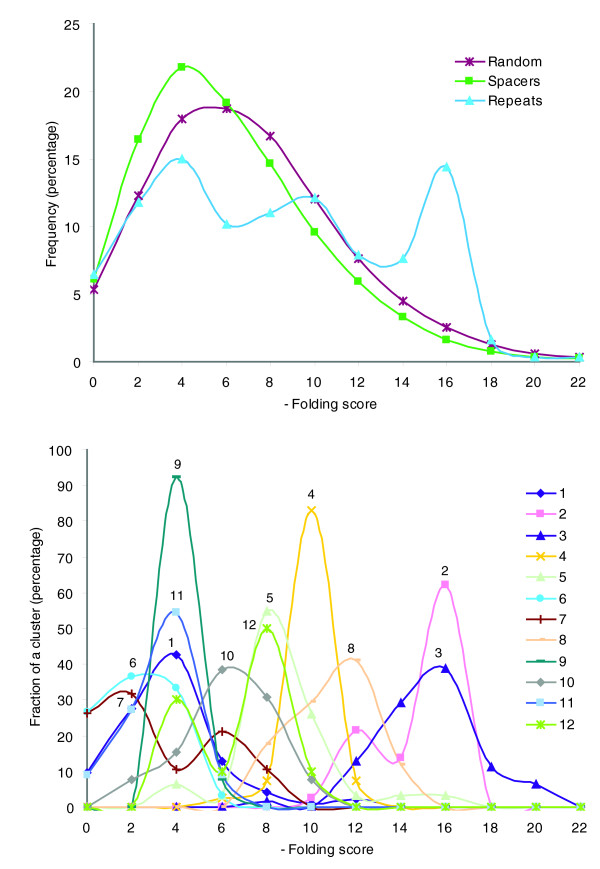
Distributions of folding scores of **(a) **all CRISPR repeats and all spacers, as compared to random sequences and **(b) **individual repeat clusters. X-axis, negative folding scores; Y-axis, fraction (percent) of total.

To assess the possibility that CRISPR repeats form stable RNA secondary structures, we used the RNAfold software [18] (see Materials and methods) to predict the intramolecular RNA structure for each of the repeats in our set. This software provides a bit-score that reflects the stability of each secondary structure. We compared the stability of the predicted secondary structure of repeats and spacers to that of similarly sized sequences selected randomly from bacterial genomes (Figure 1a). We found that the folding-score distribution of repeats deviates from the scores for random sequences, indicating a tendency of repeats to form stable secondary structure.

The trimodal pattern of the RNA folding distribution for CRISPR repeats (Figure 1a) suggests that they are not homogeneous, and that a large subset form stable secondary structures, in contrast to spacers and random sequences. To identify repeat subtypes we first attempted to align each of the 561 repeats in our set to all other repeats using the Smith-Waterman algorithm [19]. The sequence similarity results were then clustered using the MCL algorithm [20] (see Materials and methods). This procedure generated 33 clusters, 12 of which contained 10 or more members, with the largest cluster (cluster 1) containing 94 repeat sequences. Some clusters contained repeats from organisms as distantly related as Archaea and Bacteria, supporting the inference that CRISPR/CAS systems can be horizontally transferred between microorganisms [5-7].

As an independent measure for the validity of the clustering, we examined the RNA stability scores in each of the MCL-defined clusters (note that RNA stability was not taken into account in the clustering procedure). As seen in Figure 1b, clusters 2 and 3 comprise repeats with consistently high folding scores, indicating pronounced secondary structure. By contrast, clusters 1, 6, 7, 9, 10 and 11 contain repeats with consistently poor folding scores. Clusters 4, 5, 8 and 12 show intermediate folding scores, suggesting they have weaker secondary structures. Together, these groups explain the trimodal distribution observed in Figure 1a. The homogeneity of RNA structure stability scores within each cluster, along with the dramatic difference in scores between clusters, suggests that our clustering method is valid.

**Figure 2:**
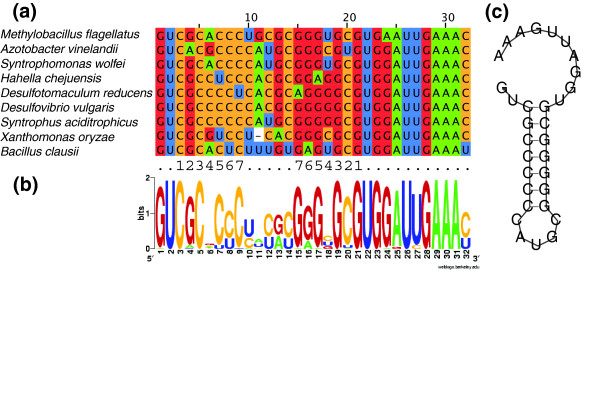
Evidence for secondary structure in cluster 3. **(a) **Multiple alignment of a subset (for clarity) of repeats in cluster 3. Numbers 1 to 7 and 7 to 1 indicate the residues involved in stem base-pairing, some compensatory mutations in the stem are highlighted with circles. Note G:U base pairing at position 5 in *Xanthomonas oryzae *and relaxed conservation of loop residues typical of RNA secondary structure in which the structure is functional rather than the sequence. **(b) **Sequence logo for all repeats in cluster 3. **(c) **Predicted secondary structure of *Syntrophus acidotrophicus *repeat using RNAfold. Stem positions are numbered in accordance with the alignment.

To further explore the observation that repeats form stable RNA secondary structures, we examined sequence alignments of the repeat clusters. CRISPR repeats are generally considered to be highly dissimilar to each other [7], except for similar repeats in strains of the same species or in closely related species [1]. However, repeats within the clusters we generated, although often containing sequences from vastly different phylogenetic groups, were generally more similar to each other and hence alignable. Figure 2a presents a multiple alignment of a subset of the repeats in cluster 3. A highly stable stem-loop structure was consistently predicted for repeats in this cluster by RNAfold [18] (Figure 1b). Notably, substitutions in the predicted stem structure are consistently accompanied by compensatory changes that preserve the base pairing (Figure 2a). This mutational pattern, together with the presence of G:U base pairs (Figure 2a), is typical of conserved RNA secondary structures and highlights the importance of the stem-loop in the repeats for the functionality of CRISPRs.

**Figure 3:**
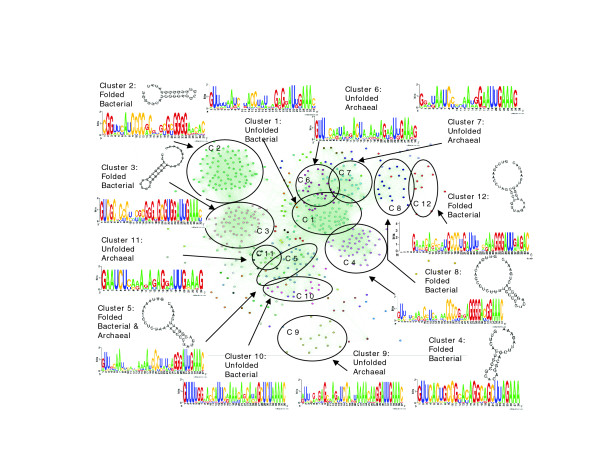
The sequence similarity space of CRISPR repeats visualized with the BioLayout (Java) program [26]. Dots denote individual repeat sequences; connecting lines represent Smith-Waterman similarities, such that closer dots represent more similar sequences. Dot colors denote cluster association as derived from MCL clustering. The 12 largest clusters are indicated by circles together with their sequence logos, coarse phylogenetic composition, and sample secondary structures where applicable.

A summary of the repeat similarity space is presented in Figure 3. As with cluster 3 (Figure 2), repeats in other clusters with high and intermediate folding scores also form stem-loop structures (Figure 3) and display compensatory mutations, suggesting stable structures. While the stem-loop motif is seen in all of these clusters, the actual sequence, as well as the length of the stem, its position relative to the unstructured region, and the size of the unstructured sequence varies between clusters. For example, while the stem in cluster 4 is typically 5 bp long and is found in the middle of the repeat, the stem in cluster 3 is typically 7 bp long, and is found towards the 5' end of the repeat (Figures 2 and 3). The difference in calculated folding scores between clusters with high and intermediate scores is likely to be due to the stem length and the frequency of GC as opposed to AT base pairings. Consistent with previous reports [7], many repeat clusters have a conserved 3' terminus of GAAA(C/G), possibly acting as a binding site for one of the conserved CAS proteins.

Two recent studies identified between 20 and 45 gene families of CASs [5,6]. Based on the tendency of CAS genes to appear together, Haft *et al*. [5] defined eight CAS subtypes (named Ecoli, Ypest, Nmeni, Dvulg, Tneap, Hmari, Apern and Mtube). We sought to determine whether our CRISPR repeat clusters corresponded to particular CAS subtypes. For this, we searched 20 kb of sequence flanking each side of the repeat array for CAS genes using the 45 CAS families TIGRFAM hidden Markov models (HMMs) defined by Haft *et al*. [5].

We found that the Ecoli CAS subtype genes appear exclusively in the proximity of structured repeat cluster 2, and, similarly, the Dvulg and Ypest CAS subtypes correspond strictly to our structured clusters 3 and 4, respectively (Table 1 and Table S1 in Additional data file 1). Presumably, specific and different sets of genes are needed in order to recognize, bind and process the different repeat types. Despite the overall pronounced correspondence between the CAS subtypes and repeat clusters, particularly for structured clusters, there are notable exceptions. For example, the reported frequent co-occurrence of the Mtube subtype with other CAS subtypes [5] is consistent with its promiscuous association with numerous repeat clusters (Table 1). Another interesting exception is the co-occurrence of the Tneap and Apern subtypes in the *Thermococcus kodakaraensis *genome with cluster 6, which is apparently due to a fusion of the Tneap and Apern subtypes (Figure S1 and Table S1 in Additional data file 1). This genome contains three CRISPR arrays, all with identical repeat sequences classified as cluster 6 (Table S1 in Additional data file 1). In some cases the CAS subtype for one or more repeat cluster members differs from the consensus for that cluster (Table S1 in Additional data file 1), suggesting that the association between CRISPR repeat subtypes and CAS subtypes is somewhat flexible.

We also identified a repeat cluster (cluster 5) that is not associated with any of the recognized CAS subtypes. We found that it is associated with most of the core CASs (cas1-4 and cas6), but lacks any of the additional type-defining genes. Cluster 5 occurs exclusively in genomes that contain other CRISPR repeat subtypes and it is possible that it employs at least part of their CAS machinery.

## Discussion

This study shows that CRISPR repeats are not structurally homogeneous and can be divided into distinct types based on sequence similarity and ability to form stable secondary structures. This explains why previous attempts to align all repeats resulted in a poorly defined consensus sequence [7]. We observed compensatory base changes in the stems of the structured repeat clusters, including G:U base pairs, indicating that the CRISPR system likely functions through an RNA intermediate.

Some clusters, such as clusters 2, 3 and 4, are discrete in the sequence similarity space, whereas the boundaries of others, such as clusters 1, 6 and 7, were not clearly defined. The discrete clusters were generally composed of structure-forming repeats, and the less well-defined clusters were composed of unstructured repeats. This may be a reflection of the greater evolutionary constraints on the stem structure.

The inference of stem-loop formation within individual CRISPR repeats is in contrast to the speculation that pairs of repeats form duplexes, and are subsequently cleaved to release spacers [6]. Such hypothesized duplexing would unlikely require the ubiquitous presence of the less conserved interior nucleotides, which would form a loop in the single repeat folding model (Figure 2) and an unpaired bulge in the duplex repeat folding model. A CRISPR array in *Sulfolobus *is transcribed and processed into 60 nucleotide long non-messenger RNAs, a size consistent with a single repeat-spacer unit [17,21], supporting the argument that transcribed spacers remain associated with their repeats. The repeats may serve to mediate contact between the spacer-targeted foreign RNA or DNA and CAS-encoded proteins. A stem-loop structure of some repeats may have evolved to facilitate recognition [22] by RNA-binding CAS-encoded proteins, although unstructured *Sulfolobus *repeats (in cluster 7; Figure 3) have been shown to bind via a sequence-specific interaction to a genus-specific protein [23]. This may partly explain the sequence conservation observed in unstructured repeats.

A previous report suggested that spacer regions contribute to the formation of secondary structures in CRISPR arrays [6]. However, we could not detect a significant deviation of spacer secondary structures from random sequences (Figure 1), indicating that spacers are unlikely to be selected based on their secondary structure. In fact, the spacers appear to have slightly weaker structures than random sequences. This is probably due to the AT richness of spacers (46% GC) relative to average bacterial genomic sequences (53% GC), as AT base pairs form less stable structures than GC pairs. The lower spacer GC content is consistent with a proposed viral origin of spacer sequences [3], as viruses are, on average, 7% lower in GC content than bacteria.

Previous attempts to classify CRISPR/CAS systems were based on CAS gene content and phylogeny (mostly of cas1) [5,6]. We add a further dimension to this classification by showing that the repeat sequence itself is also a classifying feature. This can be advantageous in instances where CRISPR arrays occur in the absence of CAS genes. For example, *Thermoplasma acidophilum *contains a CRISPR array but lacks CAS genes [5], so it cannot be classified based on CASs. Our clustering indicates that the *T*. *acidophilum *repeat belongs to (euryarchaeal) cluster 6 (Figure 3; Table S1 in Additional data file 1). In some instances, the repeat classification was able to provide higher resolution than the existing CAS classification. For example, the Nmeni subtype was reported to have an optional gene *csn2 *[5]. Our clustering divides this subtype into three clusters (10, 16 and 22). The *csn2 *gene is invariably present in one cluster (cluster 10) and absent in the other two. The finding of a repeat cluster (cluster 5) that cannot be readily resolved by associated CAS genes (see Results) further demonstrates the power of CRISPR-based classification.

The significant differences between CRISPR/CAS subtypes, both in CRISPR repeat sequence and structure, and in CAS gene content and phylogeny, raises the possibility that the subtypes also differ functionally. Support for this hypothesis could be the fact that frequently several CRISPR/CAS subtypes are found in the same genome and at least four functions have been hypothesized for these elements (host cell defense [12], regulation [5], chromosomal segregation [1] and rearrangement [11]). The study of CRISPRs is in its infancy, and their mode and function is still highly speculative. Our results provide another step toward a comprehensive understanding of these intriguing elements.

## Materials and methods

### Identification of CRISPR arrays

All genome sequences available through the IMG database version 1.50 [15] were analyzed for CRISPR arrays using the PILER-CR program [14].

### Delineation of repeat clusters

Pairwise similarities between repeats were calculated using an in-house implementation of the Smith-Waterman algorithm [19]. The best scoring similarity from the two possible repeat pair orientations, and only scores >7, were used for further analysis. Clustering of pairwise similarities was performed using the MCL program with default parameters [20]. Multiple alignments were performed using MUSCLE [24], and the alignments were manually curated, including removal of outliers. Sequence logos for each cluster were generated using WebLogo [25]. The similarity space of repeats was visualized using BioLayout (Java) [26]. The sequences of the repeats, the assignments to clusters and the multiple alignments are provided as Additional data file 1.

### Determining orientation of repeats

The PILER-CR program provides an arbitrary orientation for the repeats. To determine the correct orientation, we compared each repeat to the ones found experimentally to be transcribed into RNA [17,21], assuming that the transcribed direction is the 'correct' direction. The direction most similar to the transcribed repeats (using Waterman similarity scores [19]) was selected as the correct one. We also used the GAAA(C/G) signature at the end of some repeats in cases where the Waterman similarity scores were ambiguous. It is possible, therefore, that some repeats may be presented in the wrong orientation.

### Determination of repeat secondary structures

Structural predictions were performed using the RNA Vienna Package [27] downloaded from the Vienna Package server [28,29]. Folding scores for all repeats or individual repeat clusters were divided into bins of 2 score units and plotted as percentages. Random sequence strings with the same length distribution as repeats were generated from the analyzed genomes. The average GC contents were calculated for archaeal, bacterial and viral genomes in the IMG database, version 1.50, and the average GC content was calculated for all spacers in all genomes.

### CAS gene identification

The HMMs for CAS genes described in [5] were obtained from the TIGRFAM database, version 6.0 [30]. To identify CAS genes, all coding sequences within 20 kb of the identified CRISPR arrays were searched with the CAS HMMs using hmmpfam [31] with the thresholds of an e-value <0.001 and a positive score.

## Additional data files

The following additional data are available with the online version of this paper. Additional data file 1 contains several files showing alignments of clusters 1-12, the arrangement of the CAS cassette in the *Thermococcus kodakaraensis *genome, and CAS genes in the neighborhood of CRISPR arrays as predicted by TIGRFAM, as well as an index of organisms used in the study, a sequence fasta file containing all repeats, and a description of automatic assignment of repeats to clusters with MCL. Some files may be mac-formatted.

## Supplementary Material

Additional data file 1 Readme.txt contains a description of the files in the archive. Alignments is a directory containing manually curated fasta alignments of clusters 1-12. FigureS1.png contains a figure showing the arrangement of the CAS cassette in the *Thermococcus kodakaraensis *genome. Chromosomal coordinates are given at the top of the figure. A CRISPR array is shown to the left of the figure as red vertical lines (1 line = 5 repeats). Core CAS genes are shown in black, Apern subtype genes are shown in blue and Tneap subtype genes in red as predicted by TIGRFAM analysis (see Materials and methods). TableS1.xls is an excel-formated table containing CAS genes in the neighborhood of CRISPR arrays, as predicted by TIGRFAM (see Materials and methods). Core and type-specific genes are indicated, each genome is given both with its full name and an IMG accession code. IMG gene OIDs are given for each protein. Organisms.index is a table containing an index of organisms used in the study. Repeats.fasta is a sequence fasta file containing all repeats. Repeats.mcl describes automatic assignment of repeats to clusters with MCL. Each line contains a cluster number followed by space-separated member repeats. Some files may be mac-formatted. Click here for file

## Figures and Tables

**Table 1:** Occurrence of CAS subtypes in the proximity (± 20 kb) of the 12 largest repeat clusters

	Repeat cluster											
	
CAS subtype	1	2	3	4	5	6	7	8	9	10	11	12
Ecoli		X										
Ypest				X								
Nmeni										X		
Dvulg			X									
Tneap	X					X						
Hmari	X								X			
Apern						F	X				X	
Mtube	X					X		X				X

CAS subtypes are as defined in [5]. Associations are indicated by an X. An instance of a putative fusion between two CAS subtypes is indicated by an F.
