# Supplementary figures and images for: Evolutionary conservation of sequence and secondary structures in CRISPR repeats
Source: Genome Biol. 2007 Apr 18;8(4):R61. doi: 10.1186/gb-2007-8-4-r61 (PMC1896005; doi:10.1186/gb-2007-8-4-r61)

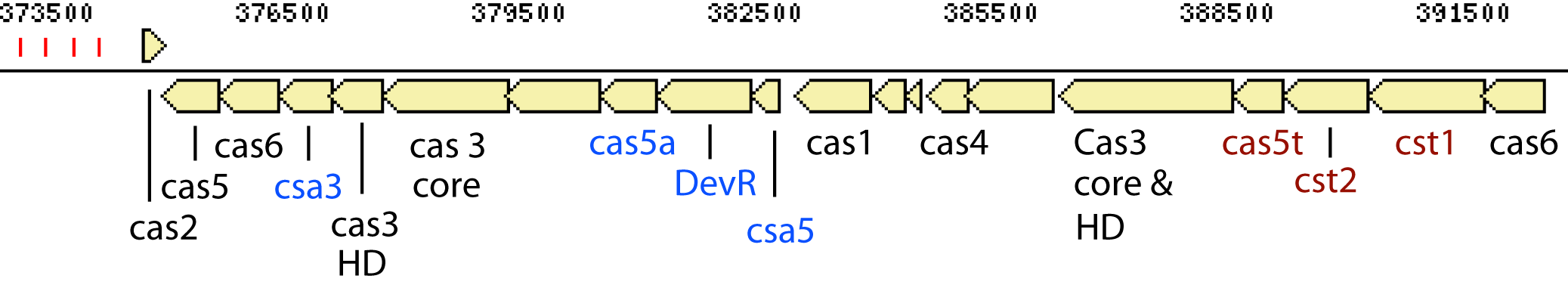

Supplement: Additional data file 1 — Readme.txt contains a description of the files in the archive. Alignments is a directory containing manually curated fasta alignments of clusters 1-12. FigureS1.png contains a figure showing the arrangement of the CAS cassette in the Thermococcus kodakaraensis genome. Chromosomal coordinates are given at the top of the figure. A CRISPR array is shown to the left of the figure as red vertical lines (1 line = 5 repeats). Core CAS genes are shown in black, Apern subtype genes are shown in blue and Tneap subtype genes in red as predicted by TIGRFAM analysis (see Materials and methods). TableS1.xls is an excel-formated table containing CAS genes in the neighborhood of CRISPR arrays, as predicted by TIGRFAM (see Materials and methods). Core and type-specific genes are indicated, each genome is given both with its full name and an IMG accession code. IMG gene OIDs are given for each protein. Organisms.index is a table containing an index of organisms used in the study. Repeats.fasta is a sequence fasta file containing all repeats. Repeats.mcl describes automatic assignment of repeats to clusters with MCL. Each line contains a cluster number followed by space-separated member repeats. Some files may be mac-formatted. [file gb-2007-8-4-r61-S1.gz › som/figureS1.png]
